# Pancreatic Cancer with Concomitant Median Arcuate Ligament Syndrome Requiring Arterial Reconstruction During Pancreaticoduodenectomy: A Case Report

**DOI:** 10.70352/scrj.cr.26-0185

**Published:** 2026-08-26

**Authors:** Wataru Miyoshino, Teijiro Hirashita, Shun Nakamura, Yuiko Nagasawa, Masahiro Kawamura, Hiroomi Takayama, Yoko Kawano, Takashi Masuda, Yuichi Endo, Masafumi Inomata

**Affiliations:** Department of Gastroenterological and Pediatric Surgery, Oita University Hospital, Yufu, Oita, Japan

**Keywords:** pancreatoduodenectomy, pancreatic cancer, median arcuate ligament syndrome, vascular reconstruction

## Abstract

**INTRODUCTION:**

Pancreatoduodenectomy (PD) for pancreatic head malignancies can be complicated by coexisting vascular anomalies. Median arcuate ligament syndrome (MALS), occurring in 2.1%–7.6% of PD cases, causes celiac artery (CA) stenosis and poses a significant risk of ischemic complications. While division of the median arcuate ligament (MAL) may restore blood flow in some cases, arterial reconstruction is occasionally necessary. We report a challenging case of pancreatic head adenocarcinoma with MALS requiring emergent intraoperative arterial reconstruction.

**CASE PRESENTATION:**

A 69-year-old man presented with a 31-mm pancreatic head tumor in contact with the superior mesenteric artery (SMA) and complete CA occlusion due to MALS. Preoperative imaging revealed well-developed collateral circulation via the dorsal pancreatic artery (DPA). After 6 cycles of neoadjuvant chemotherapy with gemcitabine plus nab-paclitaxel, the tumor decreased to 18 mm. Anticipating the potential need for arterial reconstruction during PD, preoperative simulations were conducted with the vascular surgery team. Intraoperatively, MAL division was performed, and a gastroduodenal artery (GDA) clamp test confirmed adequate hepatic arterial flow via the DPA. However, hepatic arterial flow ceased during subsequent surgical manipulation, requiring emergent arterial reconstruction. Initial middle colic artery-GDA anastomosis was complicated by repeated thrombosis; definitive reconstruction using a lesser saphenous vein graft successfully restored hepatic arterial flow. Pathological examination revealed a complete pathological response. The patient was discharged on POD day 18 without major complications and remains recurrence-free at 22 months.

**CONCLUSIONS:**

This case demonstrates that in PD for patients with concomitant MALS, collateral circulation may be disrupted by intraoperative manipulation, potentially necessitating emergent arterial reconstruction. Therefore, meticulous preoperative imaging evaluation, vigilant intraoperative monitoring of hepatic arterial flow, and thorough preparation for vascular reconstruction are essential for the safe management of these complex cases.

## Abbreviations

CA
: celiac artery
CA19-9
: carbohydrate antigen 19-9
CEA
: carcinoembryonic antigen
DPA
: dorsal pancreatic artery
GDA
: gastroduodenal artery
J1A
: first jejunal artery
J2A
: second jejunal artery
MAL
: median arcuate ligament
MALS
: median arcuate ligament syndrome
MCA
: middle colic artery
PD
: pancreatoduodenectomy
SMA
: superior mesenteric artery

## INTRODUCTION

PD is the standard surgical treatment for resectable malignant tumors of the pancreatic head.^1,2)^ However, anatomical variations and coexisting vascular lesions can significantly complicate the procedure. In particular, CA stenosis is a clinically important condition that may compromise arterial inflow to critical upper abdominal organs, including the liver and stomach, potentially resulting in ischemic complications during PD.^3–5)^ MALS is a condition in which the MAL, a fibrous structure connecting the diaphragmatic crura, compresses the CA when positioned at a low level, thereby causing impaired blood flow in the CA.^6)^ MALS has been reported to account for half of the cases of CA stenosis.^7)^ Preoperative evaluation for MALS is essential in PD, as inadequate recognition and management may lead to ischemic complications. Treatment options include surgical division of the MAL and endovascular interventions^8–10)^; however, these approaches may fail to restore sufficient blood flow in some cases, necessitating arterial reconstruction. Herein, we report a challenging case of pancreatic head adenocarcinoma with coexisting MALS requiring arterial reconstruction during PD.

## CASE PRESENTATION

**Fig. 1:**
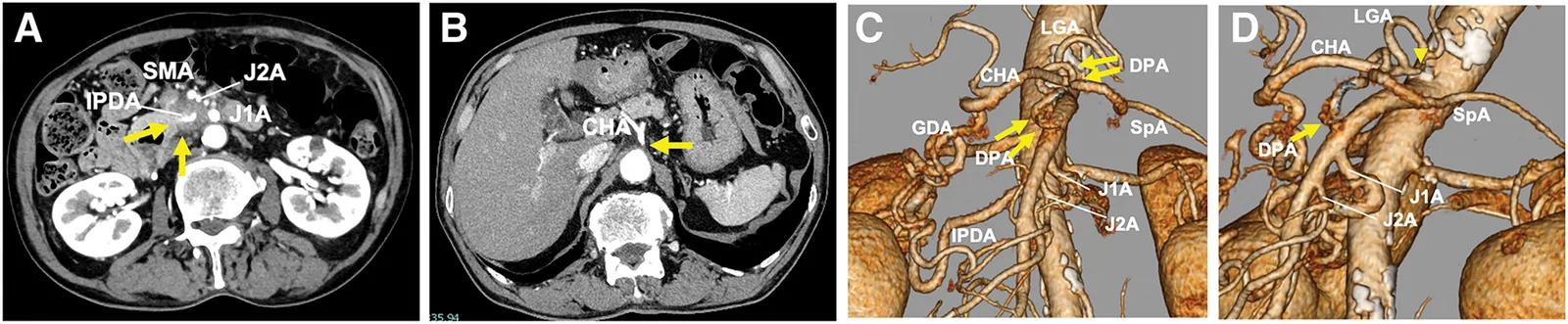
(**A**) Contrast-enhanced abdominal CT revealed a 31-mm tumor in the pancreatic head (yellow arrow) abutting the SMA and involving the J1A and J2A. (**B**) CA occlusion due to MAL compression (yellow arrow). (**C**) 3D CT angiography images demonstrating the vascular anatomy from a frontal view. The DPA (yellow arrow) originates from the anterior surface of the SMA cranial to the tumor (green), with no evidence of tumor involvement. (**D**) 3D CT angiography images demonstrating the vascular anatomy from an oblique view. CA occlusion due to MAL compression (yellow arrowhead). The origin of the DPA (yellow arrow) is distinct from the origin of the J1A. In **Fig. 1C** and **1D**, the J1A collateral artery was omitted to more clearly demonstrate the course of the DPA. CA, celiac artery; DPA, dorsal pancreatic artery; J1A, first jejunal artery; J2A, second jejunal artery; MAL, median arcuate ligament; SMA, superior mesenteric artery

**Fig. 2:**
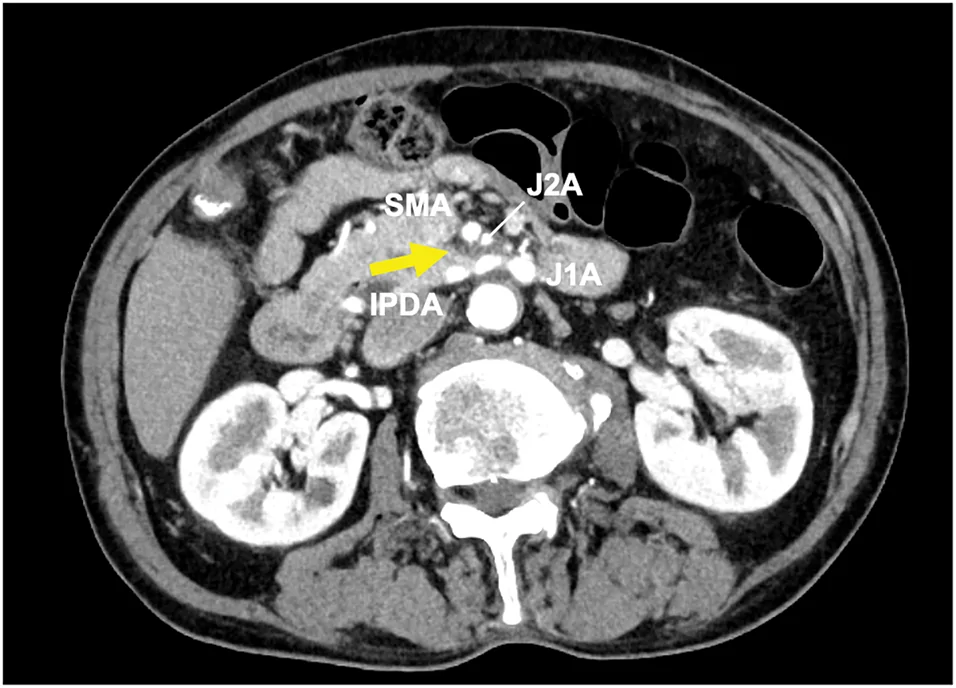
Post-chemotherapy imaging showed tumor regression to 18 mm (yellow arrow). IPDA, inferior pancreaticoduodenal artery; J1A, first jejunal artery; J2A, second jejunal artery; SMA, superior mesenteric artery

A 69-year-old man presented to our hospital with upper abdominal pain. Contrast-enhanced abdominal CT revealed a 31-mm pancreatic head tumor in contact with the SMA and invasion into the first and J1A and J2A (**Fig. 1A**). In addition, complete CA occlusion due to MALS was identified (**Fig. 1B**). To maintain CA perfusion, collateral circulation from the DPA to the splenic artery was well developed (**Fig. 1C**, **1D**). Blood chemistry tests showed no remarkable abnormalities, while tumor markers revealed a slight elevation in CEA (7.5 ng/mL), whereas (CA19-9; 29.8 U/mL) was within the normal range. Adenocarcinoma was confirmed by endoscopic US-guided fine-needle aspiration (EUS-FNA). Based on these findings, the patient was diagnosed with borderline resectable pancreatic cancer (BR-A, T3N0M0, Stage IIA). The patient underwent 6 cycles of neoadjuvant chemotherapy with gemcitabine plus nab-paclitaxel (GnP). Post-treatment imaging showed tumor shrinkage from 31 to 18 mm in diameter, and the response was evaluated as a partial response (PR) (**Fig. 2**).

**Fig. 3:**
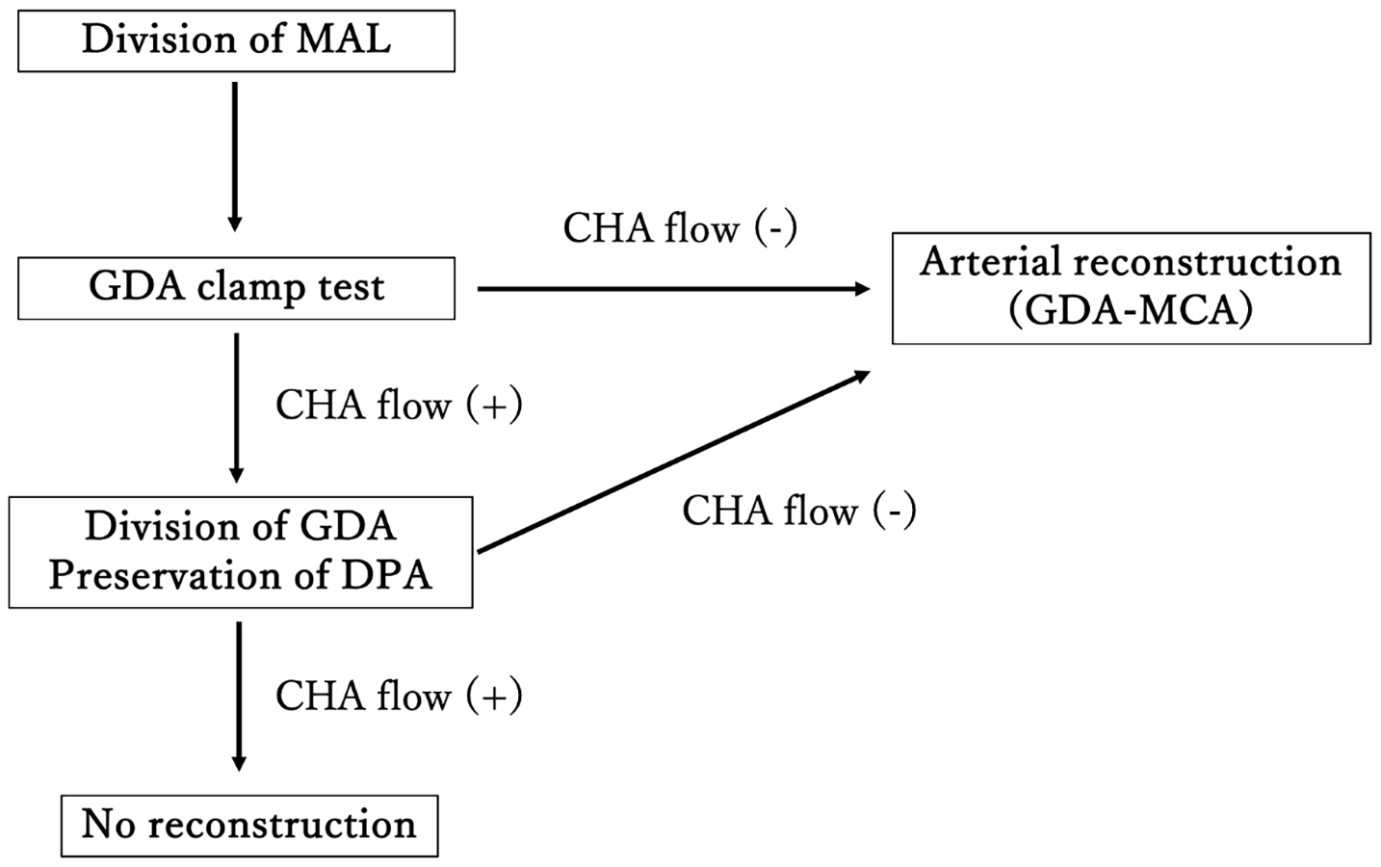
Surgical planning flowchart. CHA, common hepatic artery; DPA, dorsal pancreatic artery; GDA, gastroduodenal artery; MAL, median arcuate ligament; MCA, middle colic artery

**Fig. 4:**
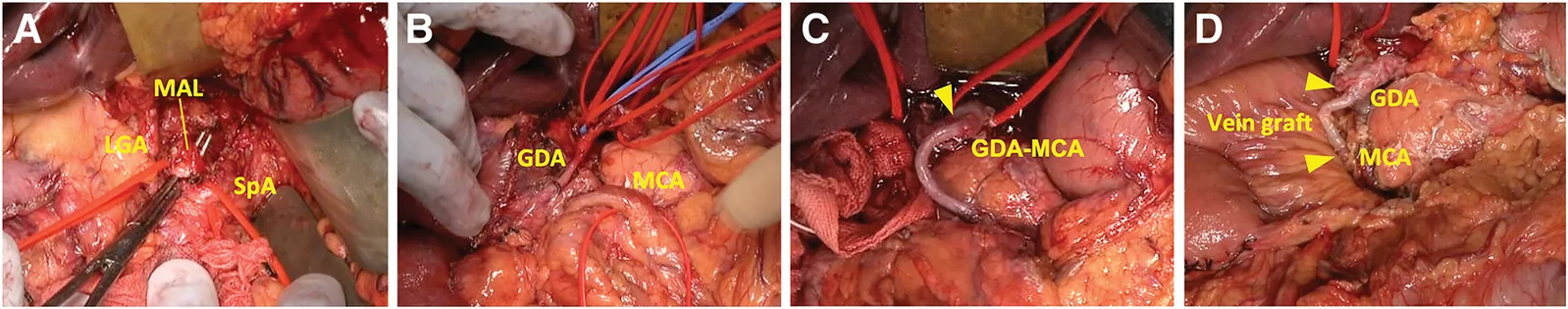
Operative findings. (**A**) The MAL was divided. (**B**) Before arterial reconstruction. (**C**) End-to-end arterial reconstruction between the MCA and the GDA was attempted; however, repeated thrombotic occlusion occurred. The anastomotic is indicated by yellow arrowhead. (**D**) Definitive reconstruction using a lesser saphenous vein graft successfully restored arterial inflow to the CA distribution. The anastomotic sites are indicated by yellow arrowheads. CA, celiac artery; GDA, gastroduodenal artery; LGA, left gastric artery; MAL, median arcuate ligament; MCA, middle colic artery; SpA, splenic artery

Based on the severity of CA occlusion, we expected limited improvement in blood flow after MAL division, preoperatively. On the other hand, although tumor invasion was observed from the dorsal aspect of the SMA extending to J1A and J2A, the DPA branched from the anterior surface of the SMA cranial to the tumor, suggesting a low likelihood of tumor involvement of the DPA, with an additional collateral artery identified between the DPA and J1A. We therefore judged that tumor resection with preservation of the DPA was feasible. Therefore, we first planned to divide the MAL and preserve the DPA. If the DPA was preserved, a GDA clamp test was performed, and PD was conducted without arterial reconstruction when hepatic arterial flow was adequate. If hepatic arterial flow was insufficient or DPA preservation was not possible, arterial reconstruction was performed using the MCA, which was free from tumor involvement. We performed surgery based on the treatment strategy shown in **Fig. 3**. We confirmed hepatic arterial flow intraoperatively by palpating the pulse of the CA and common hepatic artery (CHA). After division of the MAL, no improvement in CA blood flow was observed; however, the GDA clamp test demonstrated sufficient hepatic arterial flow, suggesting adequate perfusion via the DPA (**Fig. 4A**). Dissection of the SMA was mainly performed using a left-sided approach. The J1A was divided at its origin from the SMA. We checked the flow of the CHA repeatedly using the clamp test before dissecting the J1A and J1A collaterals, and its flow was maintained. During the final stage of dissection from the right side of the SMA, the stomach became dusky and loss of hepatic arterial flow was noted. We considered that the DPA had been unintentionally divided during dissection between the tumor and SMA, and arterial reconstruction was performed. An end-to-end anastomosis between the MCA and the GDA was attempted; however, thrombotic occlusion occurred repeatedly (**Fig. 4B**, **4C**). Ultimately, vascular reconstruction was achieved using a lesser saphenous vein graft (**Fig. 4D**). Restoration of blood flow required 6 hours. Total operative time was 11 hours and 50 minutes, with an estimated blood loss of 710 mL. To mitigate the risk of anastomotic disruption due to postoperative pancreatic fistula (POPF), the anastomosis was covered with the greater omentum and a drain was placed.

**Fig. 5:**
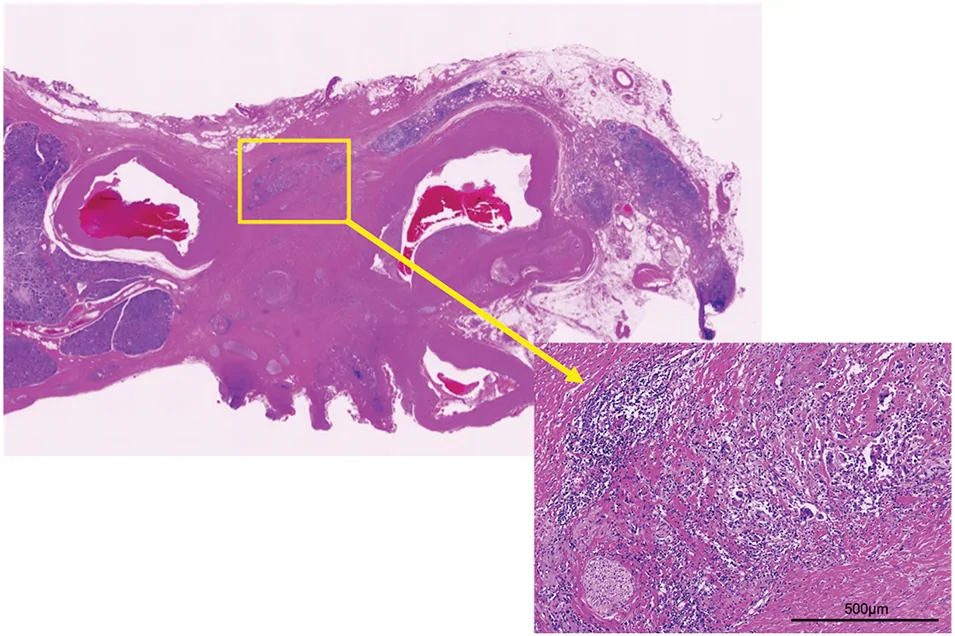
Histopathological findings. Fibrosis was observed at the site of the original tumor (hematoxylin and eosin stain). Higher magnification of the area outlined by the yellow box showing granulation tissue with a foreign-body reaction.

**Fig. 6:**
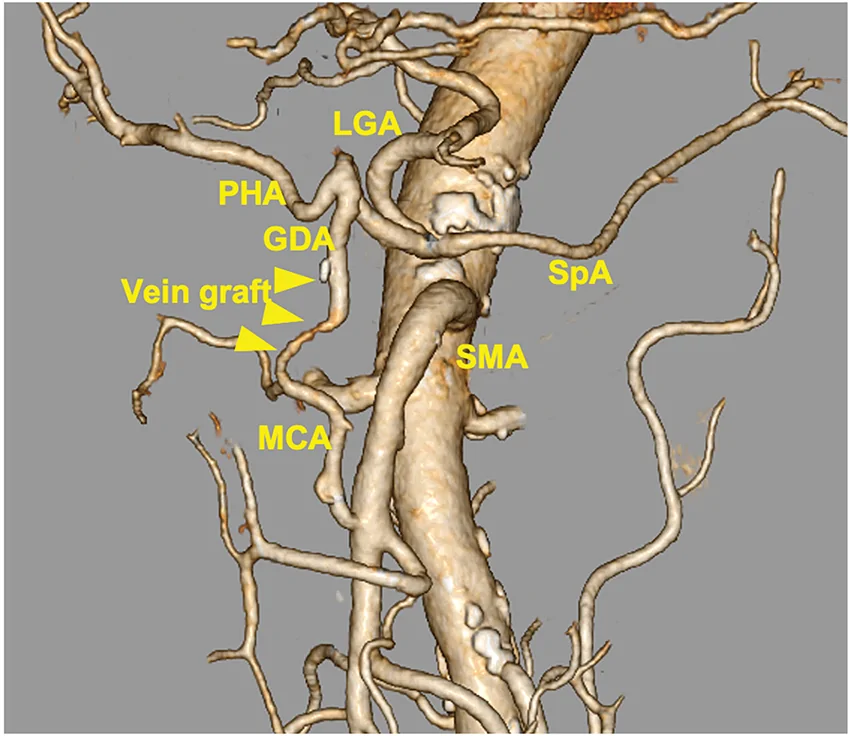
3D CT angiography on POD 7. The vein graft is indicated by yellow arrowheads. Although focal poor enhancement is observed in a portion of the graft, hepatic arterial flow is preserved. GDA, gastroduodenal artery; LGA, left gastric artery; MCA, middle colic artery; PHA, proper hepatic artery; SMA, superior mesenteric artery; SpA, splenic artery

Macroscopic examination of the resected specimen revealed no residual tumor tissue. Histopathological examination showed no residual malignant cells in the pancreas, with only fibrosis and foreign body reaction observed, confirming a complete pathological response (**Fig. 5**). The patient experienced no major postoperative complications and was discharged on POD 18. Postoperative contrast-enhanced CT demonstrated focal poor enhancement in a portion of the anastomosed vessels due to kinking; however, adequate perfusion was confirmed to be maintained (**Fig. 6**). Adjuvant chemotherapy with S-1 was administered for 6 months following the standard protocol. At 22 months of follow-up, the patient remains free of recurrence.

## DISCUSSION

MALS is an important comorbidity in patients undergoing PD, with a reported prevalence of 2.1%–7.6%.^11–13)^ PD complicated by MALS and CA stenosis has been associated with postoperative complications, including gastric dysfunction or necrosis,^4)^ postoperative pancreatic fistula (POPF),^5)^ bile leakage,^14)^ hepatic dysfunction,^15)^ and liver abscess.^16)^ Some reports have suggested that impaired blood flow correlates with the risk of postoperative complications.^5)^ Although management strategies depend on the severity of CA stenosis, release of the stenosis by division of the MAL and preservation of collateral circulation from the SMA are considered crucial.^5,8,11)^ However, in the present case, the tumor was located close to the SMA, and we anticipated from the outset that preservation of the well-developed collateral pathway via the DPA would be challenging; therefore, the surgical strategy was planned with the possibility of vascular reconstruction.

In PD for patients with concomitant MALS, some cases can be completed successfully with division of the MAL alone, which improves CA blood flow; however, in other cases, MAL division alone fails to resolve impaired blood flow, necessitating vascular reconstruction.^17,18)^ In a review by Nawara et al.,^19)^ 127 cases of PD with CA stenosis were reviewed, and it was reported that arterial reconstruction was required in 31 cases (24.4%). The reconstruction strategies were diverse, with reconstruction from the aorta being most common (15 cases), followed by the inferior pancreaticoduodenal artery (4 cases), SMA (3 cases), and MCA (2 cases). Additionally, several reports have described cases in which temporary improvement in hepatic artery flow is observed after MAL division, but blood flow subsequently ceases as surgery progresses, requiring emergent arterial reconstruction or postoperative endovascular intervention.^10,16,20)^ This is thought to result from disruption of collateral circulation due to intraoperative manipulation. In the present case, as the DPA was not considered to be involved by the tumor, we planned to complete the surgery while preserving the DPA by confirming that hepatic arterial flow was maintained throughout the procedure. Hepatic arterial flow was confirmed to be maintained through the dissection of SMA branches, including the J1A and the inferior pancreaticoduodenal artery (IPDA), and subsequent GDA transection. At this stage, DPA flow was also presumed to be intact. However, during the right-sided approach to the SMA that followed, cessation of hepatic arterial flow was detected. We therefore concluded that unintentional division of the DPA and collateral vessels had occurred at some point during this phase, most likely resulting in the loss of hepatic arterial flow. Although the tumor had shrunk after chemotherapy, peritumoral fibrosis remained; thus, during dissection aimed at achieving an R0 resection, the DPA was likely divided. In addition, the complex vascular anatomy in this case made it difficult to anticipate all collateral vessels contributing to DPA perfusion, which likely contributed to the unintentional compromise of the DPA during surgery. These findings suggest that in PD with concomitant MALS, there is always a risk of hepatic arterial flow interruption, and careful intraoperative monitoring of hepatic arterial flow is essential.

In this case, unexpected division of the DPA was identified intraoperatively, necessitating emergent arterial reconstruction. The MCA was selected as the first-choice inflow vessel because of its adequate diameter, accessibility, and ability to provide sufficient arterial inflow for rapid reconstruction. The initial end-to-end MCA-GDA anastomosis was complicated by repeated thrombotic occlusion, and definitive reconstruction was ultimately achieved using a lesser saphenous vein graft. This experience suggests that in PD, meticulous preoperative imaging assessment, careful intraoperative vascular manipulation, and adequate preparation for vascular reconstruction are critical for patients with concomitant MALS. Arterial reconstruction was a critical determinant of therapeutic success in this case. Although the reconstruction was prolonged due to repeated thrombotic occlusion during the procedure, revascularization was ultimately successfully completed using a lesser saphenous vein graft. As a result, the patient had an uneventful postoperative course.

## CONCLUSIONS

We experienced a case requiring arterial reconstruction during PD for pancreatic head cancer with concomitant MALS. In PD with concomitant MALS, careful preoperative planning and preparation for arterial reconstruction should always be considered.
